# Hereditary Influence, Animal and Human
*In deference to the complimentary wishes expressed by the writer of this able article, it is the intention of the Editor of the Psychological Journal to consider at length, in a future number, the important subject of hereditary influences.


**Published:** 1857-04-01

**Authors:** 


### HEREDITARY INFLUENCE, ANIMAL AND HUMAN.*

*(From the "Westminster Review.")*

The problem of hereditary transmission, physical and moral, although one of the most interesting of physiological problems, is also one of the most baffling. In spite of its obscurity, it fascinates the inquirer; perhaps with all the greater force because of its obscurity, for, as Spinoza truly says, men cease to admire that which they fancy they understand: *tum enim vulgus rem aliquam se satis intelligere existimat quum ipsam non admiratur.* The question of hereditary influence has descended from antiquity encumbered with prejudices and deceptive facts, which seemed coercive and conclusive, but were in truth only one-sided; and encumbered still more with hypotheses formed in ignorance of Nature's processes. It has reached us a problem still; every scientific mind not prepossessed by an hypothesis, nor content to disregard a mass of facts, must pronounce the answers hitherto proposed deficient in the primary requisite of comprehending all the phenomena. Nevertheless, answers abound. Every cattle-breeder, who rises to the height of a theory, has his theory on this complex matter, and acts upon it in the breeding of cattle and poultry. Every village gossip, every Mrs. Gamp, has her facts and her opinions, which, in expansive moments, she delivers with great confidence. Every physician has his theory, especially with reference to the transmission of disease. Even the man of letters is not without his generalization on the transmission of genius: "all men of genius," he tells you, "have had remarkable mothers;" in support of which generalization he counts off upon his fingers the illustrations which occur to him, perfectly heedless of the mass of cases in which the mothers have not been remarkable.

The various theories imply variety of interest in the question, and a practical need for the solution. A subject at once so interesting and important may well claim some attention from us here; and we shall endeavour to disengage it from all technical difficulties, so as to present it in a form intelligible to the general reader, and to clear up many misconceptions, popular and scientific, which at present obstruct the question. Dr. Lucas has in two bulky octavos gathered from far and wide a mass of material, good, bad, and indifferent, with laudable diligence, but with a want of discrimination not so laudable. He is erudite, but he has *les défauts de sa qualité*. His erudition is utterly uncritical;

* In deference to the complimentary wishes expressed by the writer of this able article, it is the intention of the Editor of the Psychological Journal to consider at length, in a future number, the important subject of hereditary influences.

and yet it is obvious that the sole value of the cases collected depends on their authenticity. It is the common error of erudite men to imagine that quantity supplies the place of quality. They fancy themselves rich when their purses are filled with forged notes; and so long as these notes are kept from presentation at the Bank, their delusion is untroubled. Dr. Lucas has far too many of these notes in his purse: the reader must take up his volumes with great caution. Mr. Orton makes no such erudite display; but he has collected some curious facts, both from his own experience and from the experience of other breeders. M. Girou is one of the authorities most frequently referred to by writers on this topic. To vast practical experience in cattle-breeding he adds very considerable physiological knowledge and force of intellect.

Heritage (*l'hérédité*), or the transmission of physical and mental qualities from parents to offspring, is one of those general facts of Nature which lie patent to universal observation. Children resemble their parents. Were this law not constant, there could be no constancy of Species: the horse might engender an elephant, the squirrel might be the progeny of a lioness, the tadpole of a tapir. The law, however, is constant. During thousands of years the offspring has continued to exhibit the structure, the instincts, and all the characteristics of the parents. Every day some one exclaims—as if the fact surprised him—"That boy is the very image of his father!" yet no one exclaims, "How like that pug dog is to its parent!" Boys or pug dogs, all children resemble their parents. We do not allude to the fact out of any abstract predilection for truisms, but simply to marshal into due prominence an important truth, on which the whole discussion of heritage must rest. The truth is this: Constancy in the transmission of structure and character from parent to offspring, is a law of Nature.

That this truth is not a truism we shall show by at once contradicting, or at least qualifying it. The very same experience which guarantees the constancy, also teaches, and with almost equal emphasis, that this constancy is not absolute. Variations occur. Children sometimes do *not* resemble their parents; which accounts for the exclamations of surprise when they do resemble them. Nay, the children are sometimes not only unlike their parents, they are, in important characteristics, unlike their Species. We then call them Deformities or Monsters, because, while their Species is distinguished by having four legs, they themselves have six or none; while their Species possesses a complex brain, they are brainless, or have imperfect brains; while their Species is known by its cloven hoofs, they have solid hoofs, and so on.* Dissemblances as great are observable in moral characteristics. We see animals of ordinary aptitudes engender offspring sometimes remarkable for their fine qualities, and sometimes for their imbecility. The savage wolf brings forth occasionally a docile, amiable cub; the man of genius owns a blockhead for his son. In the same family we observe striking differences in stature, aspect, and disposition. Brothers brought up together in the same nursery, and under the same tutor, will differ as much from each other as they differ from the first person they meet. From Cain and Abel down to the brothers Buonaparte, the striking opposition of characters in families has been a theme for rhetoric. Nor is this all. In cases where the consanguinity may be said to be so much nearer than that of ordinary brotherhood, namely, in twins, we see the same diversity; and this diversity is exhibited in those rare cases where the twins have *only one body between them.* The celebrated twins Rita and Christina† were so *fused* together, that they had

* Flachsland rapporte que deux époux bien constitutés mirent au monde trois enfans sans avant-bras ni jambes; d'autres dont parle Schmucker n'eurent que des enfans munis de douze orteils et douze doigts."—Burdach, Traité de Physiologie, ii. 264.

† See Geoffroy St. Hilaire, Philosophie Anatomique, vol. ii.; and Serres, Recherches d'Anatomie Transcendante.

only two legs between them: two legs and four arms and two heads; yet they were quite different in disposition. The same difference was manifested in the celebrated Presburg twins, and in the African twins recently exhibited in London.

It is clear, then, that offspring do not always closely resemble parents; and it is further clear, from the diversities in families, that they do not resemble them in equal degrees. Two brothers may be very unlike each other, and yet both like their parents; but the resemblance to the parents must, in this case, be variable. So that when we lay down the rule of *constancy in transmission*, we must put a rider on it, to the effect that this Constancy is not absolute, but is accompanied by a law of Variation. It is the intervention of this law which makes hereditary influence a problem; without it, heritage would be as absolute as the union of acids with bases.

Some philosophers have tried to explain the law of constancy in transmission, and its independence of the law of variation, by maintaining that it is the Species only, not the Individual, which is reproduced. Thus a sheep is always and everywhere a sheep, a man a man, reproducing the *specific type*, but not necessarily reproducing any individual peculiarities. All sheep resemble each other, and all men resemble each other, because they all belong to specific types. What does the reader say to this hypothesis? Burdach, who adopts it,* adduces his facts: for example, a dog from whom the spleen was extirpated reproduced dogs with perfect spleens; an otter, deprived of its fore paws, produced six young with their legs quite perfect; in a word, "l'idée de l'espèce se reproduit dans le fruit et lui donne des organes qui manquaient au père ou à la mère." The hypothesis has seemed convincing to the majority of thinkers, but it labours under one fatal objection—namely, Species cannot reproduce itself, for Species does not exist. It is an entity, an abstract idea, not a concrete fact. It is a fiction of the understanding, not an object existing in Nature. The *thing* Species no more exists than the thing Goodness or the thing Whiteness. Nature only knows individuals. A collection of individuals so closely resembling each other as all sheep resemble each other, are conveniently classed under one general term, named Species; but this general term has no objective existence; the abstract or typical sheep, apart from all concrete individuals, has no existence out of our systems. Whenever an individual sheep is born, it is the offspring of two individual sheep, whose structures and dispositions it reproduces; it is not the offspring of an abstract idea; it does not come into being at the bidding of a Type, which as a Species sits apart, regulating ovine phenomena. The facts of dissemblance between offspring and parents we shall explain by-and-by; they do not plead in favour of Species, because Species is a figment of philosophy, not a fact. The sooner we disengage our Zoology from all such lingering remains of old Metaphysics the better. Nothing but dreary confusion and word-splitting can come of our admitting them. Think of the hot and unwise controversies respecting "transmutation of species," which would have been spared if a clear conception of the meaning of Species had been steadily held before the disputants, or if the laws which regulate heritage had been duly considered. In one sense, transmutation of Species is a contradiction in terms. To ask if one species can produce another—*i.e.*, a cat produce a monkey—is to ask if the offspring do not inherit the organization of their parents. We know they do. We cannot conceive it otherwise. But the laws of heritage place the dispute in something of a clearer light, for they teach us that "Species" is constant, because individuals reproduce individuals closely resembling them, which is the meaning of "Species," and they also teach us, that individuals reproduce individuals *varying* in structure from themselves, which Varieties, becoming transmitted as part and parcel of the parental influence, will, in time, become so great as to constitute a difference

* Physiologie, ii. 245.

in Species. It is in vain that the upholders of "fixity of Species" assert, that all the varieties observed are differences of *degree* only. Differences of degree become differences of kind, when the gap is widened: ice and steam are only differences of degree; but they are equivalent to differences of kind. If, therefore, "transmutation of Species" is absurd, "fixity of Species" is not a whit less so. That which does not exist, can neither be transmuted nor maintained in fixity. Only individuals exist; they resemble their parents, and they differ from their parents. Out of these resemblances we create Species, out of these differences we create Varieties; we do so as conveniences of classification, and then believe in the reality of our own figments.

"Les espèces," said Buffon, boldly, "sont les seuls êtres de la nature," and thousands have firmly believed this absurdity. The very latest work published on this subject,* reproduces the dictum, and elaborately endeavours to demonstrate it. "Les espèces sont les formes primitives de la nature. Les individus n'en sont que des représentations, des copies." This was very well for Plato; but for a biologist of the nineteenth century to hold such language shows a want of philosophic culture. A cursory survey of the facts should have shown the error of the conception, if nothing else would. Facts plainly tell us that the individual and the individual's peculiarities, not those of the abstract Type, are transmitted. Plutarch speaks of a family in Thebes, every member of which was born with the mark of a spear-head on his body; and although Plutarch is not a good authority for such a fact, we may accept this because it belongs to a class of well-authenticated cases. An Italian family had the same sort of mark, and hence bore the name of *Lansada*. Haller cites the case of the Bentivoglie family, in whom a slight external tumour was transmitted from father to son, which always swelled when the atmosphere was moist. Again, the Roman families *Nasones*, and *Buccones*, indicate analogous peculiarities; to which may be added the well-known "Austrian lip" and "Bourbon nose." All the Barons de Vessins were said to have a peculiar mark between their shoulders; and by means of such a mark, La Tour Landry discovered the posthumous legitimate son of the Baron de Vessins in a London shoemaker's apprentice. Such cases might be received with an incredulous smile if they did not belong to a series of indisputable facts noticed in the breeding of animals. Every breeder knows that the colours of the parents are inherited, that the spots are repeated, such as the patch over the bull-terrier's eye, and the white legs of a horse or cow; and Chambon† lays it down, as a principle derived from experience, that by choosing the parents you can produce *any* spots you please. Girou noticed that his Swiss cow, white, spotted with red, gave five calves, four of which repeated exactly the spots of their mother, the fifth, a cow-calf, resembling the bull. And do we not all know how successful our cattle breeders have been in directing the fat to those parts of the organism where gourmandise desires it? Have not sheep become moving cylinders of fat and wool, merely because fat and wool were needed?

Still more striking are the facts of *accidents* becoming hereditary. A superb stallion, son of *Le Glorieux*, who came from the Pompadour stables, became blind from disease: all his children became blind before they were three years old. Burdach cites the case of a woman who nearly died from hæmorrhage after blood-letting; her daughter was so sensitive, that a violent hæmorrhage would follow even a trifling scratch; she, in turn, transmitted this peculiarity to her son. Horses marked during successive generations with red-hot iron in the same place, transmit the visible traces of such marks to their colts. A dog had her hinder parts paralysed for several days by a blow; six of her seven

* Cours de Physiologie Comparée, par M. Flourens, 1856. A feeble and inaccurate book.

† Traité de l'Education des Moutons, i. 116.

pups were deformed or excessively weak in their hinder parts, and were drowned as useless.* Treviranus† cites Blumenbach's case of a man whose little finger was crushed and twisted, by an accident to his right hand: his sons inherited right hands with the little finger distorted. These cases are the more surprising, because our daily experience also tells us that accidental defects are *not* transmitted; for many years it has been the custom to cut the ears and tails of terriers, and yet terrier pups do not inherit the pointed ears and short tails of their parents; for centuries men have lost arms and legs, without affecting the limbs of our species. Although, therefore, the deformities and defects of the parent may be inherited, in general they are not. For our present argument it is enough that they are so *sometimes*.

Idiosyncrasies assuredly belong to the individual, not to the species; otherwise they would not be idiosyncrasies. Parents with an unconquerable aversion to animal food, have transmitted that aversion; and parents, with the horrible propensity for human flesh, have transmitted the propensity to children brought up away from them under all social restraints. Zimmermann cites the case of a whole family upon whom coffee acted like opium, while opium had no sensible effect whatever on them; and Dr. Lucas knows a family upon whom the slightest dose of calomel produces violent nervous tremblings. Every physician knows how both predisposition to and absolute protection against certain specific diseases are transmitted. In many families the teeth and hair fall out before the ordinary time, no matter what hygiène be followed. Sir Henry Holland remarks, "the frequency of blindness as an hereditary affection is well known, whether occurring from cataract or other diseases of the parts concerned in vision. The most remarkable of the many examples known to me, is that of a family where four out of five children, otherwise healthy, became totally blind from amaurosis about the age of twelve; the vision having been gradually impaired up to this time. What adds to the singularity of this case is the existence of some family monument long prior in date, where a female ancestor is represented with several children around her, the inscription recording that all the number were blind."‡ But not only are structural peculiarities transmitted, we see even queer tricks of manner descending to the children. The writer had a puppy, taken from its mother at six weeks old, who although never taught "to beg" (an accomplishment his mother had been taught), spontaneously took to begging for everything he wanted, when about seven or eight months old: he would beg for food, beg to be let out of the room, and one day was found opposite a rabbit-hutch begging for the rabbits. Unless we are to suppose all these cases simple coincidences, we must admit individual heritage; but the doctrine of probabilities will not permit us to suppose them coincident. Let us take the idiosyncrasy of cannibalism, which may be safely said not to appear more than once in ten thousand human beings; if, therefore, we take one in ten thousand as a ratio, the chances against any man manifesting the propensity will be ten thousand to one, but the chances against his son also manifesting it will be—what some more learned calculator must declare.

Not the Species, but the Individual, then, we are forced to admit, presides over heritage; and this will help to explain many puzzling phenomena. Thus M. Danney made experiments during ten years with rabbits, a hundred couples being selected by him with a view to the creation of peculiarities. By always choosing the parents "d'après des circonstances individuelles fixes et toujours les mêmes dans certaines lignées," he succeeded in obtaining a number of malformations according to his preconceived plan. And such experiments have been repeated on dogs, pigeons, and poultry with like success. It is on this fact of individual heritage that longevity depends. There is no term of life for

* Girou, p. 127. † Biologie, iii. 452.

‡ Medical Notes and Reflections, p. 23.

the "species," only a term for the individual; a fact which sets all the speculations of Cornaro, Hufeland, and Flourens at nought. There are limits which neither the "species" nor the individual can be said to pass; no man has been known to live two hundred years; but the number of years which each individual will reach, without accident, is a term depending neither on the "species," nor on his own mode of life, but on the organization inherited from his parents. Temperance, sobriety, and chastity, however desirable, both in themselves and in their effects, will not ensure long life; intemperance, hardship, and irregularity will not prevent a man living for a century and a half. The facts are there to prove both propositions. Longevity is an inheritance. Like talent, it may be cultivated; like talent, it may be perverted; but it exists independent of all cultivation, and no cultivation will create it. Some men have a talent for long life.

M. Charles Lejoncourt published, in 1842, his *Galerie des Centenaires*, in which may be read a curious list of examples proving the hereditary nature of longevity. In one page we have a day-labourer dying at the age of 108, his father lived to 104, his grandfather to 108, and his daughter then living had reached 80. In another we have a saddler whose grandfather died at 112, his father at 113, and he himself at 115; this man, aged 113, was asked by Louis XIV. what he had done to so prolong life; his answer was—"Sire, since I was fifty I have acted upon two principles; I have shut my heart and opened my wine-cellar." M. Lejoncourt also mentions a woman then living aged 150, whose father died at 124, and whose uncle at 113. But the most surprising of the cases cited by Lucas is that of Jean Golembiewski, a Pole, who in 1846 was still living, aged 102, having been eighty years as common soldier, in thirty-five campaigns under Napoleon, and having even survived the terrible Russian campaign, in spite of five wounds, and a soldier's recklessness of life. His father died aged 121, and his grandfather 130. Indeed, the practice of every annuity and insurance office suffices to convince us of ordinary experience having discovered that length of life is somehow dependent on hereditary influence.

Although instincts, in the general acceptation of the term, may be said to belong to the species and to be transmitted with their specific type, we have abundant evidence of the individual transmission of what are called instinctive peculiarities, or acquired habits. Thus Girou relates the case of a sporting dog, taken young from its mother and father, who was singularly obstinate, and exhibited the greatest terror at every explosion of the gun, which always excites the ardour of the species. On the owner expressing his surprise to the gentleman from whom he received the dog, he was told that nothing was more likely, for the dog's father had the same peculiarity. How the vicious disposition of horses is transmitted all breeders know. Again, we know that the vice of drunkenness is very apt to be inherited; and that the passion for gambling is little less so. "A lady with whom I was very intimate," relates Da Gama Machado, "and who possessed great wealth, passed her nights in gaming; she died young, from pulmonary disease. Her eldest son was equally addicted to play, and he also died of consumption at the same age as his mother. His daughter inherited the same passion and the same disease."* Other and more crapulous vices are inherited, and are exhibited in cases where the early death of the parents, or the removal of the children in infancy, prevents the idea of any imitation or effect of education being the cause. That the "thieving propensity" is transmitted from father to son, through generations, all acquainted with police-courts know. Gall† has cited some striking examples; and that murder, like talent, runs in families, is too notorious to need illustrations here. Dogs taught to "point" or "set" transmit the talent.

* Théorie des Ressemblances, p. 154, quoted by Lucas.

† Fonctions du Cerveau, i. 207.

The American dogs inherit the peculiar cunning necessary to hunt the peccari without danger. F. Cuvier has observed that young foxes, in those parts of the country where traps are set, manifest much more prudence than even the old foxes in districts where they are less persecuted. Again, birds born in a country inhabited by man inherit their alarm at his presence; but travellers narrate that the same species encountered on uninhabited islands manifested no alarm, and are knocked down as easily as a gentleman in Fleet-street; they soon, however, learn to dread man, and this dread they transmit. As these last illustrations may be relegated to the vague region of instincts, we will confine ourselves to more individual and accidental characteristics. Thus Girou relates how a man known to him had the habit of sleeping on his back, with his right leg crossed over the left; one of his daughters showed the same peculiarity from her birth, and constantly assumed it in her cradle, in spite of her swathings. Venette knew a woman who limped with the right leg; her daughter was born with the same defect in her right leg. Ambrose Paré noticed that several children who had a peculiar mode of shaking the head, inherited it from their parents.

The inevitable conclusion from all these facts is, that parents transmit their individual peculiarities of colour, form, longevity, idiosyncrasy, &c., to their offspring, and that they do this *not as reproducing the species*, but as reproducing *their own individual organizations*. But now comes the difficult part of our inquiry:—Which is the predominating influence, that of the male, or that of the female? If both parents join to form the child, does one parent give one group of organs, and another parent another group; or do both give all?

"Half is his, and half is thine: it will be worthy of the two!"

sings the poet; and the physiologist asks,—*Which* half?

Speaking of mules, Vicq-d'Azyr says, with proper caution, that "it seems as if the exterior and the extremities were modified by the father, and that the viscera emanate from the mother." The reserve with which the great anatomist expresses himself has not been imitated by his successors; indeed, men are generally averse to uncertainties—they like a decisive opinion, a distinct formula. Hence we have the very popular formula adopted by Mr. Orton in his "Lectures"—"That the male gives the external configuration, or in other words, the locomotive organs; while the female gives the internal, or in other words, the vital organs;" which is generally stated with more scientific precision thus—"the male gives the animal system, the female the organic or vegetative." Very great and authoritative names may be cited in support of this view; and as all such formulas are the expressions of numerous facts, we must expect to find their advocates powerful in facts to support them. If there are facts which are equally explicit and diametrically opposed to those used as evidence for the theory, it is clear that the theory expresses only part of the truth. Let us see how the case stands.

Linnæus says that the *internal* plant (*i. e.*, the organs of fructification) in all hybrids is like the female; the *external* (organs of vegetation), on the contrary, resembles the male. This is, however, diametrically opposed by De Candolle, who announces it as a general law that the organs of vegetation are given by the female, those of fructification by the male.* When two doctors of such importance differ on a point like this, we may suspect that both are right and both are wrong; and here our suspicion is supported by the mass of facts adduced by the experiments of M. Sagaret,† which refute the hypothesis of Linnæus and the hypothesis of De Candolle. What we have just indicated with regard to plants, has been the course pursued with regard to animals: one class of observations has seemed to prove that the father bestows the "animal system;"

* Physiologie Végétale, p. 716.

† Pomologie Physiologique, p. 555, *sq.*

another class of observations has seemed to prove that the mother bestows it; and a third class has proved both theories inadequate. Quite recently General Daumus published the result of his long experience with Arab horses,* arguing that, according to the testimony of the Arabs, the stallion was the most valuable for the purposes of breeding. Upon this, the *Inspecteur des Haras*, who had traversed Asia for the express purpose of collecting evidence on the subject, published his diametrically opposite conclusion, declaring that it was the mare whose influence preponderated in the foal. General Daumas replied, and cited a letter addressed to him by Abd-el-Kader, who may certainly be said to understand Arab horses better than Europeans. The letter is worth reading for its own sake; we can, however, only quote its testimony on the particular point now under discussion. "The experience of centuries has established," he says, "that the essential parts of the organization, such as the bones, the tendons, the nerves, and the veins, are always derived from the stallion. The mare may give the colour and some resemblance to her structure, but the principal qualities are due to the stallion." This is very weighty testimony, on which we will only for the present remark, that it merely asserts the *preponderance* of the male influence as respects the locomotive system; it does not assert that absolute independence of any female influence maintained in the formula of Prevost and Daumas, Lallemand and others, which we are now combating. Abd-el-Kader's statement is tantamount to that made by Mr. Orton,—

"I do not mean it to be inferred that either parent gives either set of organs uninfluenced by the other parent; but merely that the leading characteristics and qualties of both sets of qualities are due to the male on the one side, and to the female on the other, the opposite parent modifying them only."

This is a much more acceptable theory than the other, but it is only an approximation to the truth. Mr. Orton's first illustration is the hybrid of the horse and ass.

"It is known that the produce of the male ass and the mare is a mule; but I do not think it is equally well-known that the produce of the stallion and the female ass is what has been denominated a hinny—yet such is the case. . . . . The mule, the produce of the ass and mare, is essentially a modified ass—the ears are those of an ass somewhat shortened—the mane is that of an ass—the tail is that of an ass—the skin and colour are those of an ass somewhat modified—the legs are slender, the hoofs high, narrow, and contracted, like those of an ass. The body and barrel are round and full, in which it differs from the ass and resembles the mare.

This description is accurate, but—we put it interrogatively—is it *always* the description of a mule, and *never* that of a hinny? This latter, the produce of the stallion and the female ass, "is essentially a modified horse—the ears like those of a horse somewhat lengthened—the mane flowing—the tail bushy like that of a horse—the skin is fine like that of a horse—the legs are stronger, and the hoofs broad and expanded like those of a horse. The body and barrel are flat and narrow, in which it differs from the horse, and resembles its mother the ass." From these facts, Mr. Orton deduces the conclusion, that the offspring of a cross is not simply a mixture of the two parents, nor is it an animal that has accidentally a similitude to one or other of its parents, inasmuch as we can produce at will either the hinny or the mule. The reader will presently see why such a conclusion cannot be accepted; and we may at once anticipate what will hereafter be more fully explained, by saying that the differences Mr. Orton signalizes are easily interpreted by another theory. In point of fact, both mule and hinny are modified asses: in each the structure and disposition of the ass predomi-

* Les Chevaux de Sahara; see also an article in the Revue des Deux Mondes, May, 1855, on Le Cheval de Guerre.

nates; and it does so in virtue of that greater "potency of race" which belongs to the ass—a potency which is less effective on the hinny, because the superior vigour of the stallion modifies it, according to ascertained laws.

"I would call your consideration," Mr. Orton continues, "to a very curious circumstance pertaining to the voice of the mule and the hinny; to which my attention was called by Mr. Lort. The mule *brays*, the hinny *neighs*. The why and wherefore of this is a perfect mystery, until we come to apply the knowlege afforded us by the law I have given. The male gives the locomotive organs, and the muscles are amongst these; the muscles are the organs which modulate the voice of the animal; the mule has the muscular structure of its sire the ass, and brays; the hinny has the muscular structure of its sire the horse, and neighs."

This seems decisive, until we extend our observations, and then we find the law altogther at fault. Thus the produce of a bull and a mare neither *lowed* nor *neighed*, but uttered a shrill cry somewhat like that of the goat. The produce of a dog and a she-wolf sometimes bark and sometimes howl, according to Buffon; and the produce of a bitch-fox and a dog, according to Burdach, barked like a dog, though somewhat hoarsely, and howled like a wolf when it was hurt. A similar remark has been made by all who have attended to cross-breeding in birds; the hybrid of the goldfinch and the canary has the song of the goldfinch mingled with occasional notes of the canary, which seem perpetually about to gain the predominance. Finally, we know how, in the human family, a magnificent voice is inherited from a mother as often as from a father.

These illustrations, apart from their interest, teach us to be cautious in generalizing from a few facts, however striking, in questions so complex as all biological questions are. Let us, however, continue to call on Mr. Orton for facts. He quotes a letter from Dr. George Wilson (whose opinion on any subject will be worth hearing) to Dr. Harvey, respecting the produce of the Manx cat and the common cat. The Manx cat has no tail, and is particularly long in the hinder legs. "You will see," says Dr. Wilson, "from the facts communicated, that where the Manx cat was the mother, the kittens had tails of a sort; where the Manx cat was the father, three-fourths of the kittens had no tail." Mr. Orton also quotes a communication made to him by Mr. Garnett of Clitheroe:—

"From these I select those pertaining to the Muscovy duck and some hybrids produced between it and the common duck. You are aware that the Muscovy drake exceeds in a striking degree the duck in size; the drake weighing from 8 to 9¼ lbs., while the duck weighs only from 3 to 4 lbs. Hybrids produced from the Muscovy drake and common duck followed this peculiarity of the male parent as to the relative size of the male and female hybrids; the male weighing from 5 to 6 lbs., the females not half as much. On the other hand, the difference in the size of the sexes when the hybrids were the produce of the common drake and the Muscovy duck, was not apparent."

A valuable observation, certainly. Mr. Orton adds the following of his own. He placed a Cochin cock with his common hens:—

"Reasoning that if the vital organs were due to the female, then the cross between these birds (being externally Cochins and internally common hens) should lay white eggs, the secretion of the egg being a vital function. You know that the Cochin lays a chocolate-coloured egg. The half-bred did what theory said they should do—laid white eggs; and not only white eggs, but eggs also which, on the evidence of myself and family, were very inferior in taste, having lost the mellow, buttery taste of the Cochin egg."

But he has recorded another curious fact respecting this same experiment, which might have made him aware of the problematical nature of his theory, had not his sagacity been hoodwinked by the theory:—

"These same half-bred birds afforded another and a very unlooked-for illustration of the position we have taken. They were all, when first hatched, like the Cochin cock, profusely feathered on the legs and feet, so much so, that they had to be marked to distinguish them from the pure bred birds. We see here that, according to the law, the male parent implanted its characteristics; but what was curious, in a few weeks, in some of the half-breeds all, and in many most, of the leg feathers were shed. Two out of some twenty birds only retained them in any very conspicuous degree. Now, why was this? The cock had implanted his external characteristics, the hen had given her vital organs. The feathers of the male were there; but the vital organs necessary to their growth were not there; and consequently, after a time, for want of nutriment, these feathers were shed."

We will not here enter on the question of the growth of feathers (a very complex matter), but, accepting his own premises, ask him, if the external characteristics are thus dependent on the vital organs for their growth and development, and these vital organs are given by the female, how does the child ever exhibit the characteristics of the male, after infancy? Of what use is it for the male to implant his characteristics, when the female influence is thus certain to annihilate them?

Mr. Orton further cites the practice of Bakewell with respect to his celebrated Dishley sheep. His rams might be bought or hired for a good price; but his best ewes were sacred. These he would neither sell nor let.

As a counter-statement, let it be noted that, according to Girou, the farmers are more particular about the bull than about the cow when they want a good milking cow, for it is observed that the property of abundant secretion of milk is more certain to be transmitted from a bull than from a cow. We question the fact of a bull having greater influence than the cow, believing that in each case the property is transmitted according to direct heritage; but that the bull should be known to have any importance in this respect, is an evidence that the "vital organs" are not solely given by the female.

The result of Mr. Orton's researches proves that the male *does* transmit his qualities to his descendants; as a matter of fact this must be always distinctly remembered; but neither his researches nor those of his predecessors suffice to prove this transmission to be *absolute*, in the sense required by those who maintain that the male gives the *animal* and the female the *vegetative* organs; as well as by those who maintain that the male influence necessarily and invariably predominates in the animal, the female in the vegetative organs. Still it is important to know that by the pollen of flowers we can modify the tints, and produce any varieties of tulip, violet, or dahlia; important to know that we can also modify the plumage of birds, and the colour of animals: it is important to know that the male qualities *are* transmissible. But for scientific rigour this is not enough. Before we can establish a law of this kind, we must be sure that the fact is constant and admits of no exceptions, or only of such apparent exceptions as may be classed under unexplained perturbations. Now daily observations, no less than recorded cases, assure us that the law is very far from being constant, that the female as unmistakeably transmits her qualities as the male transmits his, and that any theorist who should reverse the current theory and declare the mother bestowed the animal system, leaving the vegetative to the father, would be able to make a formidable array of facts. Let us glance awhile at the evidence.

It is said the male gives the colour, but the female does so likewise. A black cat and a white cat will have kittens which may be all black, all white, or black spotted with white, and white spotted with black. Every street will furnish examples. Isidore Geoffroy St. Hilaire speaks of a case under his observation, of a black buck and a white doe; the first produce was a black and white fawn; the second a fawn entirely black, except a white spot above the hoof.* Burdach mentions the case of a raven and a grey crow, who had a

* Dict. Classique d'Histoire Naturelle, x. 121.

brood of five: two black like the father; two grey like the mother; and one mixed. The same result is observed with respect to all other qualities. But perhaps the most decisive examples we could quote of the twofold influence of parents is in the singular instance recorded by Buffon. The Marquis Spontin Beaufort had a she-wolf living in his stables with a setter dog, by whom she had two cubs, a male and a female. The male resembled externally his father the dog, except that his ears were pointed and his tail like that of the wolf; the female, on the contrary, resembled her mother, the wolf, in all external characteristics *except* the tail, which was the same as her father's. Here in one case, the father gave the external characteristics, in the other the mother, while the tail was in each case, as it were, transposed. But the marvel of this case does not stop here: the cubs manifested a striking difference in disposition, in each case *resembling in character*, the parent it did *not resemble in appearance* and in sex; thus the male cub, which had all the appearance of a dog, was fierce and untameable as the wolf; the female cub, which had all the appearance of a wolf, was familiar, gentle, and caressing even to importunity. Lucas records an analogous case. These hybrids are very instructive, because the wide differences in the aspect and nature of the parents enable us to separate, as it were, the influence of each. The wolf and the dog often breed together; and the following observations, interesting in themselves, will suffice to show the reader how much caution is necessary before drawing absolute conclusions from single illustrations. Valmont Bomare observed in the various hybrids of wolf and dog which came under his notice at Chantilly, a striking preponderance of the wolf over the dog; Marsch, on the contrary, observed in his experience a preponderance of the dog over the wolf; Geoffroy St. Hilaire and Pallas found the wolf to predominate; whereas, Marolle found the cubs remarkable for their gentleness and dog-like instincts, only recalling the wolf in their voracity and fondness for flesh. Girou found the preponderance to vary; sometimes the father, sometimes the mother re-appeared in the offspring. If there were no other evidence, this would suffice to disprove the hypothesis of either parent contributing one group of organs, to the absolute exclusion of the other parent.

The same fact of twofold influence is shown in the transmission of deformities, such as extra toes, extra fingers, &c.: sometimes the male, and sometimes the female is shown to preponderate, by the offspring inheriting the deformity of the male or the female. It is well said by Girou,* that "if the organization of the male was the only one which passed to the child, the child would resemble the father, as the fruit of a graft resembles the tree from which the graft was taken, and not at all the tree on which it was grafted." And what is here said of the whole organization, applies with equal force to any one system, such as the nervous or the nutritive.

Moreover, if the hypothesis we are combating be admitted—if the father bestows the nervous system—how are we to explain the notorious inferiority of the children of great men? There is considerable exaggeration afloat on this matter, and able men have been called nullities, because they have not manifested the great talents of their fathers; but allowing for all overstatement, the palpable fact of the inferiority of the sons to their fathers is beyond dispute, and has helped to foster the idea of all great men owing their genius to their mothers, an idea which will not bear confrontation with the facts. Many men of genius have had remarkable mothers; and that one such instance could be cited is sufficient to prove the error both of the hypothesis which refers the nervous system to paternal influence, and of the hypothesis which only refers the preponderance to the paternal influence. If the male preponderates, how is it that Pericles, who "carried the weapons of Zeus upon his tongue," produced nothing better than a Paralus and a Xanthippus? How came the infamous Lysimachus from the austere Aristides? How was the weighty intellect of

* De la Génération, p. 113.

Thucydides left to be represented by an idiotic Milesias, and a stupid Stephanus? Where was the great soul of Oliver Cromwell in his son Richard? Who were the inheritors of Henry IV. and Peter the great? What were Shakspeare's children, and Milton's daughters? Unless the mother preponderated in these and similar instances, we are without an explanation; for it being proved as a law of heritage, that the individual does transmit his qualities to his offspring, it is only on the supposition of *both* individuals transmitting their organizations, and the one modifying the other, that such anomalies are conceivable. When the paternal influence is not counteracted, we see it transmitted. Hence the common remark: "talent runs in families." The proverbial phrases, "l'esprit des Mortemarts," and the "wit of the Sheridans," imply this transmission from father to son. Bernardo Tasso was a considerable poet, and his son Torquato inherited his faculties heightened by the influence of the mother. The two Herschels, the two Colmans, the Kemble family, and the Coleridges, will at once occur to the reader; but the most striking example known to us is that of the family which boasted Jean Sebastian Bach as the culminating illustration of a musical genius, which, more or less, was distributed over three hundred Bachs, the children, of course, of very various mothers.

Here a sceptical reader may be tempted to ask, how a man of genius is ever produced, if the child is always the repetition of his parents? How can two parents of ordinary capacity produce a child of extraordinary power? The answer must be postponed until we come to treat of secondary influences. For the present, we content ourselves with insisting on the conclusion to which the foregoing survey of facts has led, namely, that *both* parents are *always* represented in the offspring; and although the male influence is sometimes seen to preponderate in one direction, and the female influence in another, yet this direction is by no means constant, is often reversed, and admits of no absolute reduction to a known formula. We cannot say absolutely, "the male gives such organs;" we cannot even say, "the male always preponderates in such or such a direction." Both give all organs; sometimes one preponderates, sometimes the other. In one family we see children resembling the father, children resembling the mother, and children resembling both.

This is the conclusion inevitable on a wide survey of the facts. It is equally inevitable *à priori*, if we take our stand upon the evidence of embryology; and as some readers prefer logical deductions to any massive accumulation of facts, we will ask them to consider the question from this point of view. Reproduction, in the vegetable and animal kingdoms, is known to naturalists under three forms. In the first, a single cell spontaneously divides itself into two cells. Here it is quite clear that the child reproduces the totality of the parent. In the second form, the process called "budding" takes place: the child here grows out of the substance of the parent, until its development is completed, and then it separates from the parent to live a free life. Here also the parent is reproduced in its totality. In the third form, a higher complexity of organization has led to a more complex and more special mode of reproduction: the parent gives off from its own substance, by what may be also considered a "budding process," a mass of cells, which as pollen and ovule, as sperm-cells and germ-cells, unite to develope into plants or animals. Here again, there ought to be no doubt that the parents are reproduced; their offspring truly may be called "their own flesh and blood." Nor would the doubt have ever arisen, had not the great complexity of the organisms admitted the intervention of the Law of Variations, to which all dissemblances are due. But however such interventions may baffle our inquiries, the mind recognises at once the truth of the proposition that sperm-cell and germ-cell are as much to be regarded in the light of reproductions of the parents, as the cells produced by spontaneous division are to be regarded in the light of repetitions of the parent-cell.

And here we may glance at an ingenious hypothesis which would explain the fact of all our organs being double, by the concourse of both parents; so that the father would give one half, the mother the other half, the father the right, the mother the left side:* "Cette idée ferait présumer que notre corps est double, et que nous sommes composés de deux corps finis artistement adossés l'un à l'autre." The fact that all our organs are double—some primitively, others permanently—was first demonstrated by Serres, who, in his very remarkable work on transcendental anatomy,† has given a rapid outline of this *Lex Serriana*, as Meckel calls it. In consequence of this primitive duality of all organs (the single organs being those in the median line, and formed by the fusion of two originally distinct organs), "l'embryon résulte de la réunion de deux moitiés d'embryon; l'animal unique, si l'on peut s'exprimer ainsi, est le produit de deux moitiés d'animaux." Serres would not, however, give any countenance, we imagine, to the hypothesis of each half being furnished by each parent; for the hypothesis is contradicted by the facts of the perfect resemblance as well as perfect symmetry of each side, whereas if one parent only gave one side, we should see realized in life the fantastic combinations sometimes seen at masquerades, presenting us with a figure, half of which wears the dress of a man, and half of a woman; or half of an Italian bandit, and the other half of a good, peaceful shopkeeper.

It is now time that we should direct our attention to some of the perturbing causes, which mask the laws of transmission from our perfect apprehension. While proclaiming as absolute the law of individual transmission, while proclaiming that the parents are always reproduced in the offspring, we are met by the obvious fact of the offspring often exhibiting so marked a departure from their parents, being so different in form and disposition, that the law seems at fault. For instance, Gall speaks of a brood of wolf-cubs taken from their mother and brought up together; one was as gentle as a dog, the others retained the savageness of their species. We may also point to the fact of a man of genius suddenly starting up in an ordinary family; or to a thousand illustrative examples in which the law of individual transmission seems at fault. To explain these would be to have mastered the whole mystery of heritage; all that we can do is to mention some of the known perturbing influences.

Sir Everard Home mentions a striking case, which has become celebrated, of a thorough-bred English mare, who, in the year 1816, had a mule by a quagga—the mule bearing the unmistakeable quagga marks. In the years 1817, 1818, and 1823, this mare again foaled, and although she had not seen the quagga since 1816, her three foals were all marked with the curious quagga marks. Nor is this by any means an isolated case. Meckel observed similar results in the crossing of a wild boar with a domestic sow; in the first litter several had the brown bristles of the father; and in each of the sow's subsequent litters by domestic boars, some of the young ones were easily distinguished by their resemblance to the wild boar. Mr. Orton verified this fact in the cases of dogs, pigs, and poultry. Of the latter he says: "The so-called silk fowls have certain marked peculiarities—a silky, or downy plumage, a black skin and face, black bill and mouth, black legs, and dark or even black bones; they have, moreover, a fully-developed tuft on the head, five toes, and are feathered on the legs and feet." Peculiarities such as these were invaluable for the experiment. He found the produce of a silk cock with a common white hen to be "twelve or fifteen chickens, the whole of which had the black

* Brouzet: Essais sur l'Education Médicinale des Enfans. Paris, 1754. (Quoted by Lucas.)

† Précis d'Anatomie Transcendante. Paris, 1842, p. 238. Dr. Lucas is in error when he attributes to Florens the conception and demonstration of this important point. It is true Florens himself claims it in his last work, Cours de Physiologie Comparée, 1856.

skin, black mouth, and five toes of the silk cock—his external development. As to their plumage, I could only judge in the case of four, the rest having died in the downy state. Of these four, then, they have all the black skin and five toes of the silk cock, but, strange enough, while three of them have downy plumage, the other has feathers."

Besides this very remarkable perturbing influence, we must also consider the phenomenon of *atavism*, or ancestral influence, in which the child manifests striking resemblance to the grandfather or grandmother, and not to the father or mother. The fact is familiar enough to dispense with our citing examples How is it to be explained? It is to be explained on the supposition that the qualities were transmitted from the grandfather to the father, in whom they were *masked* by the presence of some antagonistic or controlling influence, and thence transmitted to the son, in whom, the antagonistic influence being withdrawn, they manifested themselves. As Longet remarks, "S'il n'y a pas héritage des caractères paternels il y a donc au moins *aptitude* à en heriter, disposition à les reproduire, et toujours cette transmission de cette aptitude à de nouveau descendants, chez lesquels ces mêmes caractères se manifesteront tôt ou tard."* Mr. Smith, let us say, has a remarkable aptitude for music; but the influence of Mrs. Smith is such that their children, inheriting her imperfect ear, manifest no musical talent whatever. These children, however, have inherited the disposition of their father in spite of its non-manifestation; and if, when they transmit what in them is latent, the influence of their wives is favourable, the grandchildren may turn out to be musically gifted. In the same way Consumption or Insanity seems to lie dormant for a generation, and in the next flashes out with the same fury as of old. Atavism is thus a phenomenon always to be borne in mind as one of the many complications of the complex problem. Very remarkable is the atavism exhibited by some of the lower animals, who bring forth young so utterly unlike themselves as to have been long mistaken for different species; while these young in their turn bring forth animals exactly like their ancestors. Here the children of one generation always resemble their grandfathers and grandmothers, and never their fathers and mothers.†

A third cause of complication is one which we propose to call "the potency of race or individual." Both father and mother transmit their organizations, but they do so in unequal degrees: the more potent predominates; just as if you mix brandy with equal amounts of water, soda water, and ginger beer, the taste of the brandy will predominate more in the water than in the soda water, more in the soda water than in the ginger beer.

According to Rush (quoted by Lucas), the Danes, intermarrying with women of the East, always produce children resembling the European type; but the converse does not hold good when Danish women intermarry with the men of the East. Klaproth observes the same in the mingling of the Caucasian and Mongolian races. Girou, after five-and-twenty years' experience in the breeding of sheep, found this "potency" destroy his calculations. He fancied that, by means of his Roussillon sheep and the Merino rams, he could sooner arrive at the fineness of wool which distinguishes the Merino, than if he coupled the Aveyron sheep with the Merino rams; but he found that the Roussillon type resisted the Merino so energetically that, after a quarter of a century of successive crossings, it still reappeared, whereas the Aveyron sheep had long ceased to be distinguishable from the Merinos. The same potency of particular species is noticeable in plants. Koelreuter is quoted by Burdach as having fecundated the *Nicotiana paniculata* with the pollen of *N. rustica;* and the hybrids thus produced were fecundated with the pollen of *N. paniculata*, but the plants resembled the *N. rustica*. On reversing this experiment, he still

* Traité de Physiologie, ii. 133.

† See Steenstrup on The Alternation of Generations; and Owen on Parthenogenesis.

found the female *N. rustica* to have the preponderance; so that, cross the species how he would, the *N. rustica* showed most potency.

But although we thus see that Race has a marked preponderance, we must also remember that it is subject to the individual variations of vigour, health, age, &c. Girou sums up his observations with this general remark: the offspring of an old male and a young female resembles the father less than the mother in proportion as the mother is more vigorous and the father more decrepit; the reverse is true of the offspring of an old female and a young male. In fact, if we consider that the offspring reproduces the organization of its parents, and, consequently, the organization of *that particular period*, we see at once that age, health, and general potency of organization, must be taken into the account of complicating causes. This also will help to explain—but not wholly explain—the great differences observable in the same family: differences of sex, of strength, and appearance. At present, however, science can only take note of it as a "perturbing influence."

Our survey of this great subject, brief though it has been, has enabled us to note four general facts, which sum up the present state of knowledge, and which must be steadily borne in mind in all inquiries into Hereditary Influence:—

1st. Heritage is constant: it is a law of organized beings that the organization of parents should be transmitted to their offspring.

2nd. The offspring directly represents both parents, and indirectly it represents its ancestors.

3rd. The offspring never represents its parents with absolute equality, although it represents them in every organ. Sometimes one parent predominates in one system, sometimes in another, sometimes in all.

4th. The causes of this predominance are various, some being connected with "potency" of race, or individual superiority in age, vigour, &c.; others being, in the present state of knowledge, not recognisable.

Leaving these facts without any hypothetical explanation for the present, let us pass on to a consideration of the meaning of the Law of Variation, which we have seen to be so perturbing an influence. Like produces like: that is the Law of Constancy. But we see it producing *unlike*, and the variation must have its cause. Development, whether taking place in a simple tissue or in the whole organism, must proximately arise from some alteration in the series of organic combinations. A cellular tissue would never develope into a nerve tissue, unless some new element were introduced into its composition. A whole dynasty of blockheads would never produce a man of genius by intermarriage with blockheads; the intermarriage must introduce "new blood." There is no chance in Nature. If two parents produce a child which is unlike them both, this child is not an accident: the unlikeness consists in the new combination of old elements. The cipher which stood before the numeral, thus 01, has been transposed, and we have 10 as the result. Nature transposes in this way. Out of several elements of carbon, hydrogen, and oxygen, in the same proportions, she will arrange substances so various as starch, gum, and sugar. We need not be surprised, then, if, with elements so complex as those of an organism, a great variety of combination is produced; and, far from marvelling because children sometimes are unlike their parents, the marvel truly is that they are ever like them.

The old theories could make nothing of these variations; they quietly ignored them. The once dominant, and still famous, theory of the "pre-existence of germs," which lingers in the popular expression of the "oak being contained in the acorn," maintained that the embryo is the animal in miniature. The early microscopists observing the gradual appearance of the organs, jumped to the conclusion that the organs pre-existed in the ovum, and were gradually unfolded to view as they became larger. Indeed, when we see an egg by no means increased, either in size or weight, suddenly open, and a full-formed chick emerge, the idea that the chick was pre-existent in that liquid mass which once constituted the egg seems plausible enough. Swammerdam and Malebranche pushed this notion to its logical conclusion, and declared that not only was the embryo a miniature of the adult, but the first created embryo of each species necessarily contained within itself all the germs of the future race; so that each generation included all subsequent generations. This is the famous *théorie de l'emboîtement*, which was advocated even by Cuvier. That Bonnet, Haller, and lesser men, should have been seduced by such a theory, is not remarkable, when we consider the state of knowledge in their days; but after C. F. Wolff, Blumenbach, and Von Baer had utterly refuted it, and replaced it by the sounder theory of epigenesis, to find Cuvier still giving it the sanction of his great name is a point to be remembered in the history of opinion. At the present day, we believe no one of any authority maintains the theory of pre-existence. The microscope plainly shows us that, at first, the embryo is *not* like the adult animal in any respect; the resemblance grows as development goes on; the presence of one organ determines the presence of another; and, in the earlier stages, we cannot tell whether the embryo is that of a fish, a reptile, a bird, or a mammal—much less what *kind* of fish, reptile, bird, or mammal. It is the immortal honour of C. F. Wolff to have demonstrated the great law of epigenesis,* by which the parts of an animal are made one *after* another, and *out* of the other; so that each organ may be considered as a secreting organ with respect to the others. Treviranus subsequently adopted this idea of each organ having, as it were, a secretory function with respect to the others; and Mr. Paget has luminously expanded it in his masterly "Lectures on Surgical Pathology."

When it was believed that animals *pre-existed* in the germs of the original parents, the difficulty of accounting for variations, such as deformities and malformations, was either ignored, referred to "Satanic agency," or eluded by the convenient supposition that deformed germs also pre-existed. Still there were troublesome facts not to be so got rid of. There were hybrids, for example. No one could say that there were pre-existent germs which were half horse and half donkey, or half wolf and half dog, or quarter wolf and three-quarters dog.

We will not, however, linger over such hypotheses, anxious as we are to glance at matters of more practical interest; among them, the very important question of hereditary *insanity*. Every one is familiar with the fact of the transmission of this terrible malady, but not every one is aware of the extraordinary resemblance sometimes manifested in the nature of the attacks and their periodical recurrence. Moreau relates the case of a man who, greatly agitated by the events of the French Revolution, shut himself up in one room, from which he never stirred during ten years; his daughter, on reaching the age at which he was attacked, fell into the same state, and could not be made to quit her apartment. Esquirol tells of a lady who in her twenty-fifth year went out of her mind after her accouchement; her daughter was afflicted in the same way, at the same age, and under the same circumstances. We cannot here afford space for more illustrations;† the two just cited will suffice to indicate the tragic fact, that insanity is not only transmissible, but may suddenly manifest itself in persons who have hitherto shown no predisposition to it. The fact forces upon every mind an awful sense of responsibility, when a parent or guardian has to decide on permitting a marriage where the "hereditary taint" exists. It is a subject which has recently been handled in four

* Theoria Generationis, 1795; and in a more popular version of the same work, Theorie von der Generation. We have never seen the first-named work; the second we can commend to philosophic readers.

† Dr. Forbes Winslow might take up this topic in his valuable Journal of Psychological Medicine with good effect.

fictions: in the "House of Raby," in Miss Jewsbury's "Constance Herbert," in Holme Lee's "Gilbert Massenger," and in Wilkie Collins's "Moncktons of Wincot Abbey." The three first named have used it not only as a tragic pivot, but as a moral lesson; and in so doing have taken the licence of fiction to promulgate very absolute moral views, upon which it is our duty to make some remarks.

These writers all assume that the transmission of the malady is inevitable, and hence they insist on the duty of renunciation. No one with the "hereditary taint" is justified in marrying. He must bear his burden; he must not compromise for selfish enjoyments the happiness of descendants. Were the problem really so simple as these writers make it, their moral conclusions would be indisputable. But artists are not bound to be physiologists, and are assuredly bad law givers in such cases. As artists, they employ their permitted licence in simplifying the problem of insanity to suit their stories; but when they transcend the limits of art, and moralize on their selected cases, placing them before the world as typical, they commit a serious error, and they teach questionable doctrine, because they teach it by means of fallacious facts. Let us be understood. If it were absolutely *certain* that a man whose family had the "hereditary taint" could not escape the terrible inheritance, the moral rule would be clear, the verdict against his marrying would be absolute. But happily this is by no means the case. The law of variation here intervenes. Vulgar observation confirms science in declaring this inheritance of insanity to be very *uncertain*. "La transmission héréditaire," says Burdach, in summing up, "ne s'étend, la plupart du temps, *qu'à quelques enfans*." In many cases the malady is not transmitted at all. That is to say, it is so neutralized by the influence of the other parent as not to manifest itself. Out of three children, two may inherit the malady—or only one—or none. Are all three children to be debarred from marriage on the chance that one or all may be affected? But the difficulty is further complicated. The three children, let us say, are perfectly healthy, passing into manhood and womanhood without once indicating any trace of the disease; suddenly, in mid-life, the disease breaks out,—for we are never certain of its non-appearance. Again, the three marry, have children, and die, without manifesting any of the fatal symptoms of the disease; yet their children may all be insane, because the law of *atavism* intervenes to frustrate calculations.

With such facts before us, consider the straits into which we are driven by the novelist's verdict. Three perfectly sane people are not to marry because there is a possibility of their one day becoming insane, or of their children inheriting the grandfather's malady. The same difficulty meets us in the case of *consumption* and *scrofula*, two diseases equally transmissible and almost as terrible. Are all the families in whom the consumptive "taint" exists to be excluded from marriage? To say so would be to make marriage a rarity, since few indeed among English families could be found in which no consumption has appeared during two generations. Such difficulties the novelist eludes. Yet in real life these difficulties must be met. For our own parts, while fully sensible of the responsibility, we frankly confess that we should hesitate before pronouncing against marriage, even when one of the lovers had already exhibited unequivocal signs of insanity or consumption. Nor is this said from any love of paradox; it is quite serious, as the reader will admit when he considers that the probability of transmission to children is very *uncertain*, and is entirely dependent on the other parent. A man with tubercles already formed may marry one woman who shall bear him children all perfectly healthy; whereas another woman would bear him children all inevitably doomed. It is entirely a question of organic combination; one parent's influence being neutralized or fostered by the influence of another. The same is true, if we take the case of a woman with tubercles marrying a healthy man.

Although everything depends on the constitution of the untainted parent, there is a further difficulty with human beings not felt with animals: we allude to affection, which does not spring up when bidden. You may pair your dogs and cattle according to theory; human beings must pair according to far other impulses. Nevertheless, the parent or physician who has to adjudicate in these delicate cases, may gain some guidance from general principles. We have seen that the predominance of one parent mainly consists in a superior potency which is derived from race, age, health, &c. Thus, a young man in whom the hereditary taint is visible might fall in love with a woman some few years his senior, who, to superiority of age, might add that of belonging to a more vigorous race. There would be scarcely any danger in such a marriage. But reverse the conditions—let the woman be younger, and of a less vigorous race, and marriage would present such probabilities of danger that every means of prevention should be employed. At the best, our judgment can be given with great hesitation, for the laws of organic combination, on which parental influence depends, are as yet wholly unknown.

We must forbear entering upon the many interesting topics which the application of the laws of heritage suggest, and conclude this paper with a glance at the influence of these laws in the development of the human race. History is one magnificent corollary on the laws of transmission. Were it not for these laws, civilization would be impossible. We inherit the acquired experience of our forefathers—their tendencies, their aptitudes, their habits, their improvements. It is because what is organically acquired becomes organically transmitted, that the brain of a European is twenty or thirty cubic inches greater than the brain of a Papuan, and that the European is born with aptitudes of which the Papuan has not the remotest indication. Mr. Herbert Spencer, in his very original and remarkable "Principles of Psychology," quotes the evidence of Lieut. Walpole, that "the Sandwich Islanders, in all the early parts of their education, are exceedingly quick, but not in the higher branches; they have excellent memories, and learn by rote with wonderful facility, but will not exert their thinking faculty;" which, as Mr. Spencer truly observes, indicates that they can receive and retain simple ideas, but are incompetent to the more complex processes of intelligence, because these have not become organized in the race. A similar fact is noticed in the Australians and Hindoos. Nor is this wide difference between them and the Europeans confined to the purely ratiocinative processes; an analogous difference is traceable in their moral conceptions. In the language of the Australians there are no words answering to our terms *justice, sin, guilt.* They have not acquired these ideas. In all savages the *sympathetic* emotions are quite rudimentary, and the horror which moves a European at the sight of cruelty would be as incomprehensible to the savage, as the terror which agitates a woman at the sight of a mouse. What we observe in the development from childhood to manhood, we also observe in the development of the human family, namely, a slow subjection of the egotistic to the sympathetic impulses. This has been overlooked, or not sufficiently appreciated, in the dispute about a moral sense. One school of thinkers has energetically denied that we are born with any moral sense; another school has energetically affirmed that we are born with it. And of the two we think the latter are nearest the truth. It is certain that we are so organized as to be powerfully affected by actions which appeal to this "moral sense," in a very different way from mere appeals to the intellect—the demonstration of abstract right and wrong will never move the mind to feel an action to be right or wrong; were it otherwise, the keenest intellects would also be the kindest and the justest. What is meant by the "moral sense" is the aptitude to be affected by actions in their moral bearings; and it is impossible to consider various individuals without perceiving that this aptitude in them varies, not according to their intellect, but according to their native tendencies in that direction. This aptitude to be so affected is a part and parcel of the heritage transmitted from forefathers. Just as the puppy pointer has inherited an aptitude to "point"—which, if it do not spontaneously manifest itself in "pointing," renders him incomparably more apt at learning it than any other dog—so also has the European boy inherited an aptitude for a certain moral life, which to the Papuan would be impossible. "Hereditary transmission," says Mr. Spencer, displayed alike in all the plants we cultivate, in all the animals we breed, and in the human race, applies not only to physical, but to psychical peculiarities. It is not simply that a modified form of constitution, produced by new habits of life, is bequeathed to future generations; but it is, that the modified nervous tendencies produced by such new habits of life are also bequeathed: and if the new habits of life become permanent, the tendencies become permanent."* As a consequence of this inheritance we have what is called national character. The Jew, whether in Poland, in Vienna, in London, or in Paris, never altogether merges his original peculiarities in that of the people among whom he dwells. He can only do this by intermarriage, which would be a mingling of his transmitted organization with that of the transmitted organization of another race. This is the mystery of what is called the "permanence of races." The Mosaic Arab preserves all the features and moral peculiarities of his race, simply because he is a descendant of that race, and not a descendant of the race in whose cities he dwells. That the Jew should preserve his Judaic character while living among Austrians or English, is little more remarkable than that the Englishman should preserve his Anglo-Saxon type while living among oxen and sheep; so long as no intermarriage takes place, no important change in the race can take place, because a race is simply the continual transmission of organisms. The Scotchman, "caught young," as Johnson wittily said, will lose some of the superficial characteristics, but will retain all the national peculiarities of his race; and so will the Irishman. "We know," says Mr. Spencer, "that there are warlike, peaceful, nomadic, maritime, hunting, commercial races—races that are independent or slavish, active or slothful; we know that many of these, if not all, have a common origin; and hence there can be no question that these varieties of disposition have been gradually induced and established in successive generations, and have become organic." This, indeed, is evident *à priori;* we have already seen that the instincts and habits, even the trifling peculiarities of an individual, have a tendency to become transmitted; and, what is true of the individual, is true of the race.†

It is owing to the transmission of incidentally-acquired characters that every great movement in human affairs achieves much more than its immediate object. It tends to cultivate the race. How could that new, unheard-of feeling for the wives, widows, and orphans of soldiers, which so honourably distinguished the war just closed, have ever risen, had not the sympathetic feelings of the race been cultivated during centuries of slow evolution? How could Englishmen manifest their sturdy political independence, their ineradicable love of liberty, so strikingly contrasted with the want of that feeling in other nations, had not our whole history been one bequeathed struggle against the encroachments of governments? It is, however, needless to continue: wherever we look in physiological, psychological, or sociological questions, we are certain to observe the operation of the laws of hereditary transmission.

* Principles of Psychology, p. 526. In this work heritage, for the first time, is made the basis of a psychological system; and we especially recommend any reader interested in the present article, to make himself acquainted with a treatise in every way so remarkable.

† M. Gosse, in a recently-published Essai sur les Déformations artificielles du Crâne (Geneva, 1855), shows that the forms artificially impressed on the skull during successive generations tend to become hereditary, and that, consequently, we must assign less value than has been hitherto assigned to those characteristics of distinct races which the forms of the skull have supplied.

### STATISTICS OF CAPITAL PUNISHMENT.

The following is a copy of a Return to an Address of the House of Commons, dated May 14th, 1846, signed, "H. Manners Sutton, Whitehall, May 22nd, 1846."

*Number of Persons Committed and Executed for each of the following Offences:—*

| Description of Offence. | During the five years ending with the last year of an execution. | | During the five years immediately following. |
|---|---|---|---|
| | Committed. | Executed. | Committed. |
| 1.—Cattle Stealing . . . . . . | 144 | 3 | 119 |
| 2.—Sheep Stealing . . . . . . | 1231 | 11 | 1320 |
| 3.—Horse Stealing . . . . . . | 990 | 37 | 966 |
| 4.—Stealing in a Dwelling House . | 834 | 9 | 875 |
| 5.—Forgery . . . . . . . . . | 296 | 17 | 331 |
| 6.—Coining . . . . . . . . . | 44 | 8 | 16 |
| 7.—Returning from Transportation . | 52 | * | 50 |
| 8.—Letter Stealing. . . . . . | 14 | 1 | 27 |
| 9.—Sacrilege . . . . . . . . . | 33 | 2 | 33 |
| 10.—Robbery . . . . . . . . . | 1829 | 17 | 1579 |
| 11.—Arson, and other wilful Burning | 391 | 42 | 183 |
| 12.—Piracy . . . . . . . . . | 52 | 2 | 4 |
| 13.—Attempts to Murder, unattended with bodily injuries, Shooting at, Stabbing, Wounding, &c. | 687 | 8 | 1111 |
| 14.—Violation, &c. . . . . . . . | 278 | 14 | 319 |
| 15.—Riot and Felony . . . . . | 215 | 6 | 68 |
| 16.—Other crimes . . . . . . | 105 | 11 | 118 |
| 17.—High Treason . . . . . . | 81 | 8 | 1 |
| Total . . . . . . . . | 7276 | 196 | 7120 |

| | During five years ending with 1832. | | During five years immediately following. | |
|---|---|---|---|---|
| | Committed. | Executed. | Committed. | Executed. |
| Burglary and House-breaking . . . | 4327 | 46 | 3734 | 3 |

The return is a statement of the crimes capital in 1830 for which the punishment of death has been abolished by statute, or for which it has not been inflicted during the last five years, giving in columns the number of persons committed and executed for each offence during five years, ending with the

* The last execution for returning from transportation was in 1810. The records in the Home Office do not show the executions for this offence in the five previous years.

The capital punishment for house-breaking was abolished in 1833, and for burglary (except when violence to persons is used) in 1837.—*Parliamentary Paper*, No. 354.

last year of an execution for it, and the commitments during the five years immediately following.

It appears, from this table, that during a period of five years, when the sentence of death was recorded and carried into effect in seventeen separate kinds of offences, there were 7276 committals and 196 executions; whereas, in the five succeeding years, in which there were no executions for any of the aforesaid offences, the committals were 7,120, making a diminution in the number of commitments on the average of 156. Nor do these figures fully represent the case. When the punishment of death for such offences as sheep-stealing, forgery, coining, and the like was in operation, prosecutors were reluctant to prefer the charge; and, consequently, the number of committals did not adequately represent the number of offenders; but when the capital sentence was repealed, this objection was removed; but, taking the figures as they appear, it is quite clear that, contrary to the predictions and arguments of those who still retain a preference for capital punishment, crime did not increase when the capital punishment was abolished.

In analysing this table, two classes of offences require remark:—1. Attempt to murder unattended with bodily injuries, shooting at, stabbing, wounding, &c.; and 2, violation, &c.; the numbers of which are respectively:—

| | During the five years ending with the last year of an execution. | | During the five years immediately following. |
|---|---|---|---|
| | Committed. | Executed. | Committed. |
| Attempts to Murder, &c. | 687 | 8 | 1111 |
| Violation . . . . . . | 278 | 14 | 319 |

In the former, where the committals rose to 1111, many were included which it had been customary in the former five years to class as committed for simple assaults, and so indicted to avoid the risk of capital conviction; so in the latter case, since the repeal of the capital penalty, juries convict upon the graver charge about double the proportion in the same number of commitments, taking the average of five years. Formerly many offenders, though capitally indicted, were convicted on the minor count, viz., "assault with intent," and in the criminal tables were recorded in this latter class of offenders, thus reducing the apparent number of commitments on the first five years on the capital charge.

This return is exceedingly interesting, and appears most completely to refute the objections of those persons who still oppose the entire abolition of capital punishment on the ground, as they say, that its abolition would increase crime.

